# Alteration in Gut Microbiota Associated with Zinc Deficiency in School-Age Children

**DOI:** 10.3390/nu14142895

**Published:** 2022-07-14

**Authors:** Xiaohui Chen, Yu Jiang, Zhuo Wang, Youhai Chen, Shihua Tang, Shuyue Wang, Li Su, Xiaodan Huang, Danfeng Long, Liang Wang, Wei Guo, Ying Zhang

**Affiliations:** 1 School of Public Health, Lanzhou University, Lanzhou 730000, China; chenxh20@lzu.edu.cn (X.C.); jiangy20@lzu.edu.cn (Y.J.); chenyh629@163.com (Y.C.); 18189579145@163.com (S.T.); sul@lzu.edu.cn (L.S.); huangxiaodan@lzu.edu.cn (X.H.); longdf@lzu.edu.cn (D.L.); 2 Scientific Research Office, Shaanxi Provincial People’s Hospital, Xi’an 710000, China; zhwang1220@163.com; 3 School of Public Health, Peking University, Beijing 100000, China; wangshuyue21@bjmu.edu.cn; 4 Department of Public Health, Robbins College of Health and Human Sciences, Baylor University, Waco, TX 76798, USA; liang_wang1@baylor.edu; 5 Key Laboratory of Animal Genetics, Breeding and Reproduction in the Plateau Mountainous Region, Ministry of Education, Guizhou University, Guiyang 550000, China

**Keywords:** zinc deficiency, gut microbiota, diet, cytochrome P450, school-age children

## Abstract

Zinc deficiency could lead to a dynamic variation in gut microbial composition and function in animals. However, how zinc deficiency affects the gut microbiome in school-age children remains unclear. The purpose of this study was to profile the dynamic shifts in the gut microbiome of school-age children with zinc deficiency, and to determine whether such shifts are associated with dietary intake. A dietary survey, anthropometric measurements, and serum tests were performed on 177 school-age children, and 67 children were selected to explore the gut microbial community using amplicon sequencing. School-age children suffered from poor dietary diversity and insufficient food and nutrient intake, and 32% of them were zinc deficient. The inflammatory cytokines significantly increased in the zinc deficiency (ZD) group compared to that in the control (CK) group (*p* < 0.05). There was no difference in beta diversity, while the Shannon index was much higher in the ZD group (*p* < 0.05). At the genus level, *Coprobacter*, *Acetivibrio*, *Paraprevotella*, and *Clostridium_XI* were more abundant in the ZD group (*p* < 0.05). A functional predictive analysis showed that the metabolism of xenobiotics by cytochrome P450 was significantly depleted in the ZD group (*p* < 0.05). In conclusion, gut microbial diversity was affected by zinc deficiency with some specific bacteria highlighted in the ZD group, which may be used as biomarkers for further clinical diagnosis of zinc deficiency.

## 1. Introduction

As a crucial digestive organ of the human body, the gastrointestinal tract contains large and diverse microbial populations [1]. Numerous studies have demonstrated that gut microbiota may contribute to the health of human hosts [2]. The intestinal microbiota has various significant functions, including defending against pathogens, enhancing the immune system, promoting metabolism, and the biosynthesis of essential amino acids and vitamins [3,4,5]. Dysbiosis of the gut microbiota is associated with numerous human diseases such as obesity, inflammatory bowel disease, non-alcoholic liver disease, and type 2 diabetes mellitus [6,7,8,9]. Therefore, the gut microbiome plays a key role in maintaining host health. Although the dominant taxa in the gut of human beings are similar, the relative abundances and strain types of certain microbial populations vary greatly across individuals [10]. In addition, the diversity of the intestinal flora may be impacted by many factors, including environment, age, antibiotics, and the host’s physiological condition [11,12]. Among these factors, the diet, which is the most easily modifiable or controlled factor, may play a vital role in regulating the stability of the intestinal flora [13]. Recently, many studies have reported that various dietary components could result in shifts of microbial composition [14,15,16,17]. Of these, micronutrients from the diet are of great importance for the human body and particularly for the resident population of gut microbes [18]. In earlier studies, several models were used to explore the effect of micronutrients on gut microbiota [19]. For example, an iron-deficient diet caused the decrease of *Bilophila* spp., *Coprococcus* spp., and *E. hallii* in germ-free rats [20]. In an in vitro colonic fermentation study, iron-deficiency reduced the growth of potentially pathogenic intestinal bacteria [21]. Moreover, dietary supplementation with copper altered the abundances of some taxa, such as *Corynebacterium*, *Rikenella*, *Jeotgailcoccus*, and *Staphylococcus* [22]. Therefore, we hypothesize that an unbalanced micronutrient intake would result in the change of the microbial structure.

Zinc homeostasis is mainly regulated by intestinal absorption [23]. A study revealed that nearly 20% of zinc intake could be utilized by gut microbiota [24]. As an essential micronutrient for the human body, zinc is involved in numerous physiological reactions [25]. For example, zinc not only plays essential roles in RNA and DNA synthesis, the immune system, and enzymatic reactions [26,27], but demonstrates potential value in preventing and treating COVID-19 [28]. In addition, Reed et al. found that zinc deficiency significantly increased the abundances of *Enterococcus* and *Ruminococcaceae* and decreased those of *Clostridiales* and *Peptostreptococcaceae*, while decreasing the microbial diversity in a broiler chicken gut [29]. It was found that acute zinc deficiency caused obvious changes on the phylum level and increased the gut permeability and inflammation response in pregnant mice [30]. Additionally, a study about Malaysians found that serum zinc level was positively associated with taxa belonging to *Clostridiales* and negatively associated with taxa belonging to *Bacteroidales* [31]. The above findings have thus suggested that zinc has the potential to regulate gut microbial communities.

About 17% of the world’s human population suffers from zinc deficiency [32]. In China, about one billion people were at high risk of zinc deficiency [33]. It has been demonstrated that an inadequate zinc intake could result in stunting and increase the risk of infection and diarrhea [26]. Moreover, several animal models demonstrated that zinc deficiency was associated with decreased gastrointestinal health and an altered microbiome [29,30,34], but shifts in the gut microbiota of humans associated with zinc deficiency have yet to be studied. Thus, the present study aims to investigate the effects of zinc deficiency on the composition and function of gut microbiota (amplicon sequencing) in school-age children, which may offer potential ways to address zinc deficiency and thus pave the way towards improving the health and nutritional status of affected individuals.

## 2. Materials and Methods

### 2.1. Recruitment of Sample Populations

Participants in this study were recruited at Lujiagou Central Primary School, Lujiagou Town, Anding District, Dingxi City, Gansu Province (104°34′ E, 35°50′ N) in November 2020. The questionnaire containing detailed information is shown in the Supplementary Text.

A total of 177 school-age children were enrolled and 67 children (26 zinc-deficient participants and 41 control participants) were chosen to explore the gut microbial community using amplicon sequencing according to the selection criteria [35], which included: (1) no history of acute or chronic illness (cancer, malignant tumors, kidney disease, heart disease, diabetes, liver disease, etc.); (2) no gastrointestinal symptoms (constipation, diarrhea, etc.) in the past three months and no history of chronic gastrointestinal diseases (gastrointestinal tumors, etc.); (3) no other chronic diseases; (4) no infectious diseases (AIDS, hepatitis b, hepatitis c, syphilis, etc.); (5) no history of intake of antibiotics, antiviral, antifungal, or analgesic drugs in the past three months; (6) no fever symptoms in the past three months; and (7) no nutritional supplements (zinc, calcium, vitamin, etc.) taken in the past three months. Informed consent has been obtained from the guardians of school-age children enrolled in this study. Registration number (ChiCTR2200056909) was obtained from Chinese Clinical Trial Registry (http://www.chictr.org.cn/index.aspx) (accessed on 1 May 2022).

### 2.2. Calculation and Verification of Sample Size

According to the previously published literature, the prevalence of zinc deficiency in Chinese children was 38.2% [36]. The formula for estimating the sample size was as follows:

(1) $$n=\frac{Z_{\alpha /2}^{2}\times p\times \left(1-p\right)}{\delta^{2}}$$

where α = 0.05, confidence level = 1 − α = 95%, Z_α/2_ = 1.96, *p* = 0.382, and *δ* is the permissible error, where *δ* = 0.2 × *p* = 0.0764. Therefore, the estimated minimum sample size was 155. 

In addition, in order to test whether the data generated in this study have enough statistical power, power analysis [37] was performed to determine it and the result showed that when the power value was 0.8 (a power 0.8 is often desired) and significant level (α) was 0.05, the estimated sample size was 25.52 per group, which was smaller than the minimum sample size (26 in ZD group) in the current study. This suggests that the sample size in the current study is sufficient for this analysis. 

### 2.3. Dietary Survey and Anthropometric Measures

Three consecutive 24-h dietary recalls were used to collect the dietary information by trained interviewers. Both chefs of school cafeteria and primary caregivers were asked to recall the food the school-age children consumed at school and home to obtain total dietary information. Entry and calculation of dietary data were conducted by the software Nutrition (V.2.7.8.8, Beijing Bowen Shixun Technology Corporation & Institute of Nutrition and Food Safety, Chinese Center for Disease Control and Prevention, Beijing, China). To obtain dietary diversity score (DDS), the nine food groups were selected according to Dietary Guidelines for Chinese Residents (2016) [38]. To assess the nutrient adequacy of study objects, the nutrient adequacy ratio (NAR) and mean adequacy ratio (MAR) were calculated. The related calculation methods followed methods that were previously reported [39].

Standard procedures were followed to measure the height and weight by trained interviewers. HAZ (height for age Z-score), BMI (body mass index), WAZ (weight for age Z-score), and BMIZ (BMI for age Z-score) were calculated by WHO Anthroplus software (V.1.0.4, Department of Nutrition, World Health Organization, Geneva, Switzerland).

### 2.4. Collection and Determination of Blood Samples

Whole blood was collected from children under fasting state. Serum was obtained after centrifugation at 3000× *g* for 5 min and stored at −80 ℃. All the procedures were conducted in accordance with proposed protocols of the International Zinc Nutrition Consultative Group (IZiNCG) [40].

After being thawed at room temperature, flame atomic absorption spectrometer (Analytik Jena, ZEEnit 700P, Jena, Germany) was used to detected serum zinc. Enzyme-linked immunosorbent assay (ELISA) kits (Elabscience, Wuhan, China) were used to detect the level of serum inflammatory cytokines IL-6, IL-1β, and TNF-α.

### 2.5. Collection and DNA Extraction of Stool Samples

About 300 mg feces were collected per person and then transferred to the laboratory on dry ice within 24 h of collection and then stored at −80 °C. According to the manufacturer’s protocol, QIAamp DNA stool mini kit (QIAGEN, Hilden, Germany) was used for DNA extraction. NanoDrop 2000 (Thermo Fisher Scientific, Waltham, MA, USA) and Qubit3.0 Fluorometer (Thermo Fisher Scientific, Waltham, MA, USA) were used to test the concentration and purity of the genomic DNA. The integrity of genomic DNA was detected by 1% agarose gel electrophoresis.

### 2.6. Polymerase Chain Reaction (PCR) and Sequencing

During the polymerase chain reaction, which was conducted with a TopTaq DNA polymerase kit (Transgen, Beijing, China), the V3–V4 regions from the bacterial 16S rDNA gene were amplified with universal primer set *341F* (5′-CCTACGGGNGGCWGCAG-3′) and *805R* (5′-GACTACHVGGGTATCTAATCC-3′) [41]. According to the manufacturer’s protocol, final PCR products were purified and quantified by Agencourt AMPureXPPCR Purification Beads (Beckman Coulter, Grants Pass, OR, USA) and Agilent 2100 bioanalyzer (Agilent Technologies, Santa Clara, CA, USA). The sequencing was performed on Illumina NovaSeq 6000 platform (2 × 250 bp) at Genesky Biotechnologies Inc. (Shanghai, China). Amplicon sequencing is the high-throughput sequencing of PCR products of specific gene segments such as 16S rDNA, 18S rDNA, and ITS [42]. Among them, the 16S rDNA sequencing used in this study is an important and widely used tool for obtaining information about microbial communities’ structures, microbial taxonomy, and community diversity [43].

### 2.7. Bioinformatic Analysis

The raw data were processed in QIIME2 [44]. Cutadapt plugin was used to trim the adaptor and primer sequences. Quality control and chimera removal were conducted by DADA2 plugin [45]. Sequences were clustered into amplicon sequence variants (ASVs). Assignments of ASV were performed by Naive Bayes classifier that was trained on the Ribosomal Database Project (V.11.5) [46]. The alpha diversity indices were calculated by R software (Vegan package, V.4.0.5, R Core Team, Vienna, Austria) [47]. Non-metric multidimensional scaling (NMDS) based on Bray–Curtis distance (Beta-diversity) was conducted to assess whether the microbial community was affected by zinc level using R software (V.4.0.5). Significantly different taxa between the two groups were determined by Linear discriminate analysis effect size (LEfSe) [48]. The bar plots were made in Origin 2021 at phylum and genus level. Pearson correlation analysis was conducted by R software (V.4.0.5) to show the potential relationship between gut microbes and dietary components. Kyoto Encyclopedia of Genes and Genomes (KEGG) functional predictive analysis was carried out by PICRUSt2 software (V.2.3.0, The Huttenhower Lab, Cambridge, MA, USA) [49].

### 2.8. Statistical Analysis

Data entry and documentation were conducted by EpiData 3.1 (EpiData Association, Odense, Denmark). DDS, intake of food, intake of energy and nutrients, NARs, MAR, alpha diversity indices, and blood indicators were presented as mean and standard error of mean (SEM), and Student’s *t* test was used to examine the significant differences between ZD group and CK group. Z score was presented as median, and Mann–Whitney U test was conducted between the two groups. GraphPad Prism 8.0 was used for statistical analysis. For low, medium, and high DDS groups, one-way analysis of variance (ANOVA) and Bonferroni test were performed to examine the differences in NARs and MAR. Pearson correlation analysis was used between NARs and DDS. Welch’s *t*-test was used to compare the relative abundances of the pathways and functional genes between two groups by STAMP software (V.2.5.3, Robert Beiko & Christian Blouin, Halifax, NS, Canada). * *p* < 0.05, ** *p* ≤ 0.01 and *** *p* ≤ 0.001 indicated the statistically significant differences in figures and tables.

## 3. Results

### 3.1. Characteristics of Study Populations

According to the serum zinc results, 177 participants were divided into the ZD and CK groups, including 57 zinc-deficient members and 120 members with normal zinc levels, respectively (the specific lower cut-offs of the serum zinc concentration were as follows [40]: 65 μg/dL for children under 10 years old, 70 μg/dL for females aged ≥ 10 years, and 74 μg/dL for males aged ≥ 10 years). The detailed information of the participants (z score, blood indices) is presented in Table 1. A total of 30 females and 27 males were included in the ZD group (8.35 ± 0.15 years; mean ± SEM), and 63 females and 57 males were enrolled in the CK group (8.88 ± 0.18 years; mean ± SEM). In the ZD group, IL-6, TNF-α, and IL-1β were much higher (*p* < 0.05).

### 3.2. The Nutritional Profiles of School-Age Children 

Cereals and potatoes, followed by milk and milk products, vegetables, fruit, and egg were the five most consumed food. By contrast, aquatic products, soybeans, and nuts were less likely to be consumed. Among the nine food groups, the intakes of vegetable, fruit, livestock and poultry meat, aquatic products, milk and milk products, soybeans and nuts, and oil were far below the recommended intake (Supplementary Table S1). The data on the daily consumption of nutrients and food for the ZD group and CK group are shown in Supplementary Table S2. Only the intake of soybeans and nuts significantly increased in the ZD group compared to that in the CK group (*p* < 0.05). The NARs and MAR were not significantly different between the two groups (Supplementary Table S3).

According to the tertiles, the DDS was divided into low, medium, and high groups (cutoff for low or high: DDS < 5.6 or DDS > 6, Table 2). Compared to a low DDS, most of the NARs were significantly higher in medium and high DDS (*p* < 0.05) with the exception of sodium and Vitamin E, and the same trend was found for the MAR. The DDS was positively correlated with all NARs and with the MAR (r > 0, *p* < 0.05).

### 3.3. Microbial Taxa Composition Varies in Different Groups 

After quality control, a total of 4,503,833 high-quality reads were obtained with an average of 67,211 ± 421 (mean ± SEM) high-quality reads per sample, which yielded 4210 ASVs with an average of 234 ± 6 ASVs per sample. The coverage indices in both groups are close to 1, suggesting that the sequencing depth is sufficient to cover the microbial community (Table 3).

**Figure 1:**
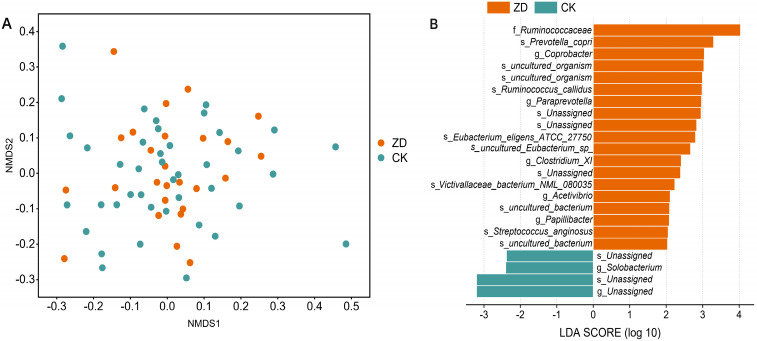
Microbial community diversity and differential taxa in ZD and CK groups. (**A**) NMDS showed β-diversity using Bray–Curtis distance. Each dot represented one sample, and two colors represented two groups, respectively. (**B**) LEfSe identifying the taxa that significantly explained differences in community composition between ZD and CK groups.

NMDS based on the Bray–Curtis distances demonstrated that no difference was observed in the bacterial community compositions between the ZD and CK groups (*p* = 0.926, PERMANOVA; Figure 1A). In the ZD group, the Shannon index was much higher (*p* < 0.01, Table 3).

**Figure 2:**
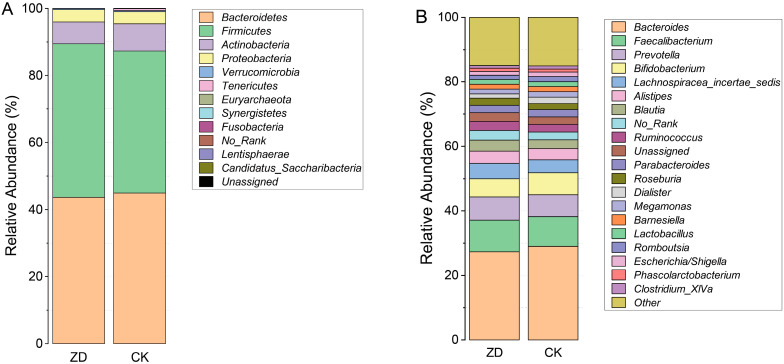
Bacterial composition between ZD and CK groups. (**A**) Microbial composition at phylum level; (**B**) Microbial composition at genus level.

At the phylum level, the four most abundant phyla were *Bacteroidetes* (44.43%), *Firmicutes* (43.70%), *Actinobacteria* (7.44%), and *Proteobacteria* (3.64%), accounting for 99.21% of the total ASVs in all samples (Figure 2A). At the genus level, *Bacteroides* (28.33%), *Faecalibacterium* (9.48%), *Prevotella* (6.91%), and *Bifidobacterium* (6.39%) were the common genera across samples, accounting for 50.11% of the total reads (Figure 2B). According to the Venn diagram, 954 ASVs were shared between the ZD group and CK group, and 1327 and 1929 ASVs were unique to the ZD group and CK group, respectively (Supplementary Figure S1).

As shown in Figure 1B, the LEfSe revealed that *Ruminococcaceae*, *Acetivibrio*, *Paraprevotella*, *Coprobacter*, *Clostridium_XI*, and *Papillibacter* were more abundant in the ZD group (LDA > 2, all *p* < 0.05), while *Solobacterium* was more abundant in the CK group (LDA = 2.404, *p* < 0.05). The differential abundance analysis was performed at the ASV level.

### 3.4. The Relationships between Microbial Taxa and Nutrition Intake

**Figure 3:**
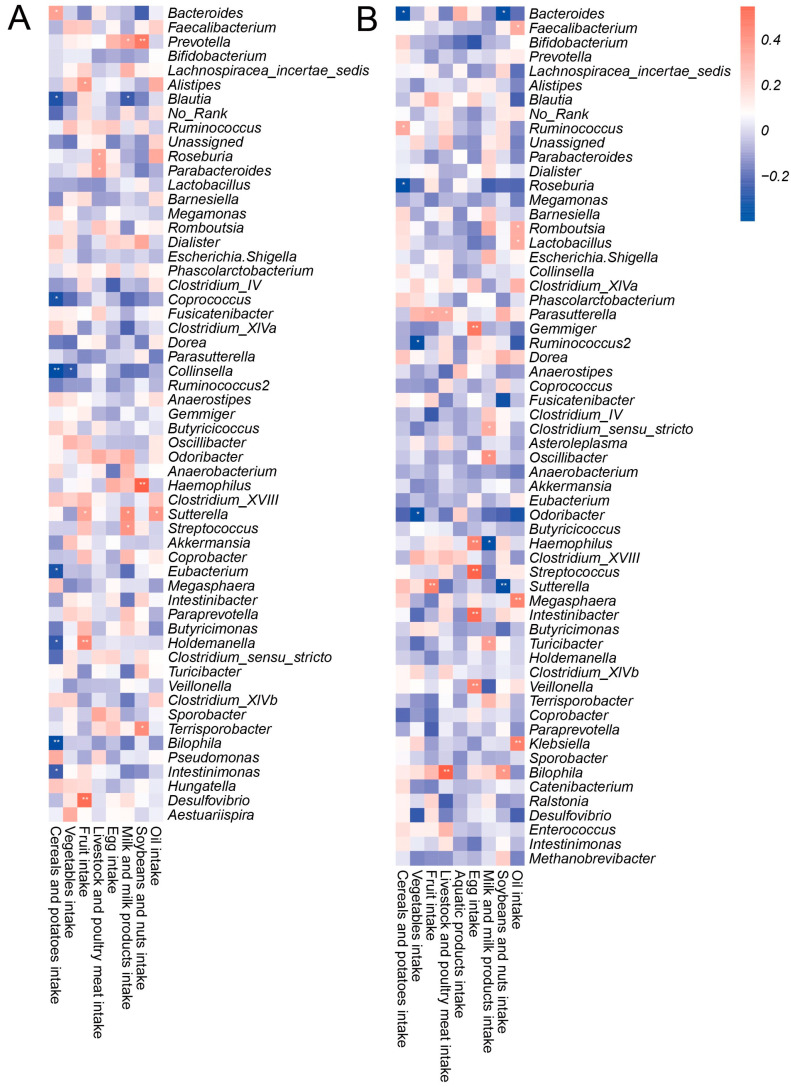
The relationship between genus-level bacterial taxa and dietary composition in (**A**) ZD group and (**B**) CK group. Only those genera with relative abundance >0.05% are shown. Two colors mean positive correlation and negative correlation, respectively.

The relationship between dietary intake and microbial taxa (genus level) was calculated by the Pearson correlation analysis, with the correlation coefficients >0.30 or <−0.30 presented in the figures. As shown in Figure 3, the intake of cereals and potatoes was positively associated with *Bacteroides* in the ZD participants (r = 0.04, *p* < 0.05), while a negative association was observed between them in the CK participants (r = −0.38, *p* < 0.05).

The nutrient intake also showed associations with some genera-level microbiota (Supplementary Figure S2). In the CK group, each nutrient exhibited significant associations with microbes. The relative abundance of *Barnesiella* was positively associated with the intake of 11 nutrients (0.31 < r < 0.48, *p* < 0.05). The relative abundances of *Bilophila* (0.31 < r < 0.54, *p* < 0.05) and *Dorea* (0.31 < r < 0.48, *p* < 0.05) were positively associated with the intake of nine and eight nutrients, respectively. In the ZD group, only nine nutrients showed significant associations with microbiota.

### 3.5. Alteration in Microbial Function between Zinc-Deficient Group and Control Group

**Figure 4:**
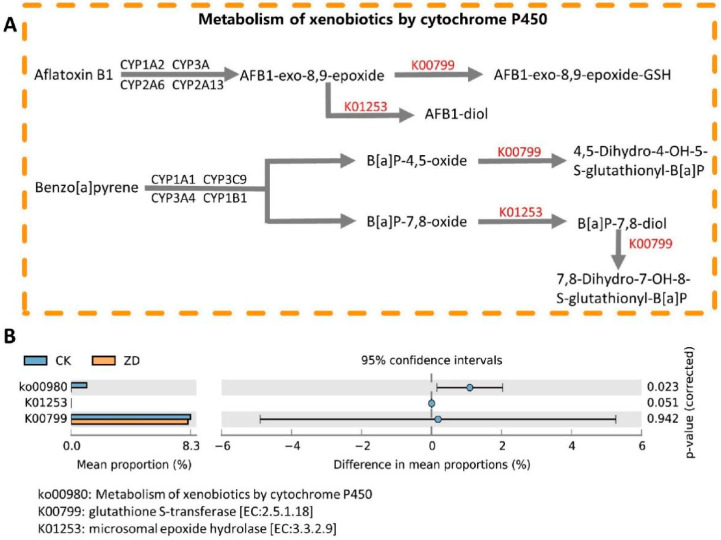
Zinc deficiency promotes distinct functional shifts of gut microbiota. (**A**) Illustration of metabolism of xenobiotics by cytochrome P450 and the related KOs. (**B**) Changes of pathway and KOs in CK and ZD groups. All numbers of KOs and pathway were obtained from KEGG database.

To explore the functional shifts in the gut microbes, a functional predictive analysis was conducted using the PICRUSt2 software (V.2.3.0, The Huttenhower Lab, Cambridge, MA, USA). A total of 166 KEGG metabolic pathways (at level 3) were obtained. The predominant pathways in both groups were the biosynthesis of ansamycins, other forms of glycan degradation, and the biosynthesis of vancomycin group antibiotics. Regarding the metabolic pathway, the metabolism of xenobiotics by cytochrome P450 was significantly depleted in the ZD group (Figure 4B, *p* < 0.05). In total, 40 KEGG orthologies (KOs) were significantly increased in the ZD group (*p* < 0.05), whereas 72 KOs were significantly decreased in the ZD group (*p* < 0.05). Most of these KOs fell into “Microbial metabolism in diverse environments” and “Metabolic pathways”. In addition, two enzymes involved in the “Metabolism of xenobiotics by cytochrome P450” pathway were observed (Figure 4A). Glutathione S-transferase (K00799) and microsomal epoxide hydrolase (K01253) were decreased in the ZD group, although no statistical significance was found (Figure 4B).

## 4. Discussion

Recently, interactions between micronutrients and intestinal microflora have attracted great interest [50]. It has been shown that microbes can affect the availability of micronutrients and the levels of micronutrients, which in turn could influence the gut microbiota [50]. For example, *Lactobacillus plantarum* increased not only iron absorption in non-heme diets [51], but also magnesium and calcium bioavailability [52]. A study on mice showed that a magnesium-deficient diet significantly decreased the gut bacterial diversity [53]. In mice models, manganese supplementation significantly increased the *Firmicutes* abundance while reducing the *Bacterodetes* abundance [54]. In this study, we explored the effects of zinc deficiency on the gut microbiome of school-age children and observed the alteration of microbial diversity and microbiome function.

As a key trace element, zinc was obtained from diets to compensate the urinal and fecal zinc losses [55]. The zinc status of the children was not only influenced by the zinc content of the food consumed but also depended on its bioavailability [56]. Vegetarians are susceptible to zinc deficiency as plants contain a major inhibitor of zinc absorption (phytate) [57]. Phytogenic phytase, which catalyzes phytic acid hydrolysis, could be activated during food processing [58]. Thus, zinc bioavailability was lower in non-refined grain diets [40]. In contrast, animal-sourced foods without zinc absorption inhibitors are excellent sources of zinc [32]. The subjects in our study were from Dingxi, a major potato growing area in China, where a large proportion of the human population grow potatoes and cereals for self-consumption. For the subjects of our study, the most frequently consumed food group was cereals and potatoes, while the consumption of aquatic products and meat was less frequent. Therefore, a high intake of unrefined plant-derived food and a low intake of animal-derived food may be one of the reasons for the high zinc deficiency rate in the current study. In addition, a poor dietary diversity may be another cause of the zinc deficiency observed in this study. The DDS was positively associated with the NARs, which was consistent with previous studies [39,59]. As a valid indicator for evaluating micronutrient deficiency, a DDS below six indicated a higher risk of micronutrient deficiency [59]. Therefore, a balanced diet would aid the prevention of zinc deficiency.

As for the relationship between the gut microbiome and dietary components, the intakes of cereals and potatoes were positively associated with *Bacteroides* in the ZD group, which showed the opposite trend in the CK group. *Bacteroides* are involved in several important metabolic activities in the human colon to obtain energy and provide nutrients for the host [60]. In general, the consumption of western diets rich in fat and protein increased *Bacteroides* abundances [61], while vegetarian diets high in fiber decreased *Bacteroides* spp in humans [62]. However, the metabolic pathways concerning “carbohydrate metabolism”, “glycan biosynthesis and metabolism”, and “nucleotide metabolism” were depleted under the low zinc status [63], which may reduce the production of energy and nutrients. Meanwhile, *Bacteroides* is reported to be able to adapt itself to dynamic environments by various mechanisms [64]. Thus, *Bacteroides* may actively utilize the cereals and potatoes consumed by the human host for carbohydrate fermentation and starch breakdown to produce energy and nutrients for its own survival [60,65] while the host is afflicted with zinc deficiency, which represents a potential compensation mechanism.

Significant alterations of the intestinal flora were observed in the ZD group. In the current study, the microbial diversity significantly increased in the ZD group. However, Zn-limited diets significantly decreased the microbial diversity in weaned pigs and broiler chickens [29,66]. A low microbial diversity was usually associated with a range of diseases in recent studies, such as asthma [67], autism [68], and allergies [69]. It was reported that a reduction in microbial diversity was likely to be a crucial but non-specific indicator of an insufficient zinc intake [29]. A possible explanation for this discrepancy concerning diversity may be attributed to the differences in the subjects. Therefore, more studies on gut microbiota in humans with zinc deficiency are needed. Another potential reason may be that micronutrient deficiency disturbs the microecological balance and promotes the bloom and colonization of certain pathogens [70], which can lead to the increase of microbial diversity. In addition, recent findings focused on the potential relationship between zinc deficiency and neurological disorders [71]. For example, a growing number of research reports have linked zinc deficiency to autism spectrum disorders (ASD) in humans, [72,73] and the dysbiosis of gut microbes was commonly found in children with ASD [74]. Similar findings were reported in previous studies, where the relative abundances of *Clostridium*, *Prevotella*, and *Ruminococcaceae* were increased in autistic children [75,76,77]. On the other hand, zinc was a key dietary component that had positive effects on gut integrity [78], and zinc deficiency affected gastro-intestinal development and increased the intestinal permeability [30,34]. The alteration in the intestinal barrier caused high levels of endotoxin entering the bloodstream, which activated inflammatory responses and elevated the levels of pro-inflammatory cytokines [30,34,79]. Consequently, it is reasonable to hypothesize that zinc deficiency may influence both neurological development and intestinal barrier function, which leads to the alteration of the microbial composition and inflammatory status.

In the current study, *Ruminococcaceae*, *Acetivibrio*, *Clostridium_XI*, and *Papillibacter* were more abundant in the ZD group. This finding agrees with those of previous studies reporting that the abundances of *Clostridium*, *Ruminococcus*, and *Ruminococcaceae* were increased in a low zinc state [29,34]. Some of these taxa were involved in the competition for zinc between the host and microbes. For example, *Ruminococcaceae* Bacterium CPB6, belonging to *Ruminococcaceae*, contained several genes encoding ABC transporters [80], among which the ZnuABC transporter was associated with the transmembrane transport of zinc ions [81]. It has been found that the ZnuABC transporter was induced in certain taxa under zinc-limiting conditions [82,83]. Thus, we speculated that the ZnuABC transporter helped bacteria plunder the dietary zinc in the lumen from the host, which directly caused the occurrence of zinc deficiency and even aggravated the condition. In addition, the phylum *Firmicutes*, containing *Ruminococcaceae*, *Acetivibrio*, *Clostridium_XI*, and *Papillibacter*, may gain survival advantages by reducing the demand for zinc or by possessing more efficient intake mechanisms in low zinc environments [30]. In addition, short chain fatty acids (SCFAs) would decrease the luminal pH, thereby increasing zinc bioavailability [84]. As SCFA producers, the increased *Ruminococcaceae* and *Clostridium* could indirectly contribute to the absorption of zinc by intestinal cells, which may be a kind of potential regulatory mechanism for maintaining zinc homeostasis. However, there is insufficient available evidence to fully explain the association between these taxa and zinc deficiency. Further studies are needed to determine the internal mechanism.

According to the results of the KEGG function prediction, the metabolism of xenobiotics by cytochrome P450 was significantly depleted in the ZD group. As a superfamily of enzymes, cytochrome P450 (CYP) participates in a variety of physiological processes in mammals, including the metabolism of drugs and xenobiotics [85] and the biosynthesis of steroid hormones [86]. A previous study on rats found that zinc deficiency could result in the down-regulated the expression of the hepatic genes *CYP4b1*, *CYP4A3*, and *CYP2C23*, which are involved in xenobiotic metabolism [87]. It was reported that zinc supplementation could enrich the metabolism of xenobiotics by cytochrome P450 and improve xenobiotic metabolism in previous animal experiments and clinical studies [88,89]. However, another study [90] found that oxidative stress caused the down-regulation of the cytochrome P450 level. Animal models have demonstrated that zinc deficiency could cause oxidative stress in liver [91,92], along with the generation of free radicals. The free radicals may result in the structural and functional impairment of liver microsomal P450 and eventually cause the decrease of enzymatic activity and metabolic capability [93]. Additionally, microsomal epoxide hydrolase and glutathione S-transferase participate in the metabolic processes of various compounds in the “metabolism of xenobiotics by cytochrome P450” pathway. Beloucif et al. noticed a remarkable depletion of glutathione S-transferase in rats with dietary zinc deficiency [94]. As a part of an antioxidant system, this enzyme would consume itself to eliminate the reactive oxygen species caused by zinc deficiency [95]. Draper et al. found that microsomal epoxide hydrolase could be inhibited by Zn^2+^ in rats and humans [96]. Yet, the serum zinc concentration was decreased while the liver zinc concentration increased during inflammation [97], which may lead to the down-regulation of CYP enzyme activity in liver. The above results have provided evidence that zinc deficiency may have a negative impact on CYP enzyme activity, thereby influencing the detoxification of xenobiotics.

## 5. Conclusions

Overall, this study linked serum inflammatory cytokines and dietary surveys with the gut microbial community to explore the effect of zinc deficiency on the composition of intestinal bacterial communities, and we found that the sample children had a high prevalence of zinc deficiency due to their inappropriate dietary patterns and poor dietary diversity statuses. Moreover, the present results suggested that zinc deficiency caused the alteration of gut microbiota and their functional capacity. However, the results from the present study were based on self-reports by the guardians or caretakers of the subjects enrolled in the dietary surveys, which may be susceptible to recall bias when estimating food intake; therefore, the present findings warrant further studies to elucidate the casual associations between zinc deficiency and the diversity and function of the gut microbiome.

## Figures and Tables

**Table 1:** Characteristics of zinc-deficient and control children (n = 177).

	ZD (n = 57)	CK (n = 120)	*p*
Age, years (mean ± SEM)	8.35 ± 0.15	8.88 ± 0.18	0.025 *
Sex (females, males)	30 females, 27 males	63 females, 57 males	0.987
Dietary Diversity score	5.71 ± 0.10	5.78 ± 0.07	0.581
Z-score			
Median HAZ score	−0.29	−0.37	0.232
Median WAZ score	0.00	−0.37	0.043 *
Median BMIZ score	−0.04	−0.20	0.121
Blood indices (mean ± SEM)			
Serum zinc (μg/dL)	18.21 ± 2.70	141.62 ± 4.23	<0.001 ***
IL-6 (pg/mL)	29.10 ± 3.32	19.00 ± 1.06	<0.001 ***
TNF-α (pg/mL)	14.38 ± 0.50	8.58 ± 0.53	<0.001 ***
IL-1β (pg/mL)	32.11 ± 4.77	22.75 ± 1.65	0.023 *

* *p* < 0.05, and *** *p* < 0.001.

**Table 2:** NARs and MAR among participants in DDS low, medium, and high groups (n = 177).

NARs	Overall		Low (n = 53)		Medium (n = 74)		High (n = 50)		*p ^2^*	*r ^3^*	*p*
	Mean	SEM	Mean	SEM	Mean	SEM	Mean	SEM			
Vitamin A	0.52	0.02	0.40 ^a^	0.03	0.54 ^b^	0.02	0.63 ^c^	0.03	<0.001 ***	0.46	<0.001 ***
Vitamin D	0.12	0.01	0.08 ^a^	0.01	0.13 ^b^	0.01	0.14 ^b^	0.01	0.001 ***	0.28	<0.001 ***
Vitamin E	0.99	0.01	0.98	0.02	0.99	0.01	1.00	0.00	0.306	0.25	0.001 ***
Vitamin B_1_	0.61	0.02	0.53 ^a^	0.03	0.64 ^b^	0.03	0.66 ^b^	0.03	0.002 **	0.25	0.001 ***
Vitamin B_2_	0.57	0.01	0.46 ^a^	0.03	0.60 ^b^	0.02	0.66 ^b^	0.02	<0.001 ***	0.41	<0.001 ***
Vitamin C	0.47	0.02	0.33 ^a^	0.03	0.47 ^b^	0.04	0.60 ^c^	0.04	<0.001 ***	0.29	<0.001 ***
Niacin	0.62	0.02	0.52 ^a^	0.03	0.64 ^b^	0.03	0.69 ^b^	0.03	<0.001 ***	0.29	<0.001 ***
Calcium	0.30	0.01	0.26 ^a^	0.02	0.31 ^b^	0.01	0.34 ^b^	0.01	<0.001 ***	0.29	<0.001 ***
Phosphorus	0.96	0.01	0.90 ^a^	0.03	0.98 ^b^	0.01	0.99 ^b^	0.00	<0.001 ***	0.34	<0.001 ***
Potassium	0.74	0.02	0.63 ^a^	0.03	0.76 ^b^	0.02	0.83 ^b^	0.02	<0.001 ***	0.35	<0.001 ***
Sodium	0.99	0.01	0.98	0.02	1.00	0.00	1.00	0.00	0.152	0.31	<0.001 ***
Magnesium	0.74	0.02	0.64 ^a^	0.03	0.76 ^b^	0.02	0.81 ^b^	0.02	<0.001 ***	0.28	<0.001 ***
Iron	0.82	0.02	0.75 ^a^	0.03	0.84 ^b^	0.02	0.88 ^b^	0.02	0.004 **	0.22	0.004 **
Zinc	0.66	0.02	0.56 ^a^	0.03	0.69 ^b^	0.02	0.74 ^b^	0.02	<0.001 ***	0.35	<0.001 ***
Selenium	0.76	0.02	0.63 ^a^	0.03	0.79 ^b^	0.02	0.84 ^b^	0.02	<0.001 ***	0.43	<0.001 ***
MAR	0.66	0.01	0.58 ^a^	0.02	0.67 ^b^	0.02	0.72 ^b^	0.01	<0.001 ***	0.40	<0.001 ***

*^2^ p* value was calculated by one-way ANOVA. *^3^* Pearson correlation coefficients (r) and *p* value were calculated. ^a,b,c^ Different lowercase letters in the same row indicated significant differences between the groups. ** *p* < 0.01, and *** *p* < 0.001.

**Table 3:** Alpha diversity indices between zinc-deficient group and control group.

	ZD		CK		*p*
	Mean	SEM	Mean	SEM	
Chao1	252.61	10.56	226.69	8.26	0.056
Shannon	4.09	0.06	3.81	0.07	0.005 **
Coverage	1.00	0.00	1.00	0.00	0.901

** *p* < 0.01.

## Data Availability

Raw sequence reads have been uploaded into European Nucleotide Archive (https://www.ebi.ac.uk/ena/browser/home), and the project number was PRJEB51060. (accessed on 20 March 2022).

## Supplementary Materials

The following are available online at https://www.mdpi.com/article/10.3390/nu14142895/s1. Supplementary Text: Questionnaire on the nutritional status of school-age children; Table S1: Average intake versus recommended intake for nine food groups; Table S2: The daily consumption of food groups and nutrients between ZD group and CK group; Table S3: Nutrient adequacy ratio of nutrients among participants by zinc statue groups; Figure S1: Venn diagram of unique and shared ASVs (amplicon sequence variants) in the ZD group and CK group; Figure S2: The relationship between genus-level bacterial taxa and nutritional profile in (A) ZD group and (B) CK group. Only those genera with relative abundance >0.05% are shown. Two colors mean positive correlation and negative correlation, respectively.
