# Supplementary material for: Alteration in Gut Microbiota Associated with Zinc Deficiency in School-Age Children
Source: Nutrients. 2022 Jul 14;14(14):2895. doi: 10.3390/nu14142895 (PMC9319427; doi:10.3390/nu14142895)
Supplement: Supplementary file 1 [file nutrients-14-02895-s001.zip › nutrients-1784098-supplementary.pdf]

Supplementary Text：Questionnaire on the Nutritional Status of School-age Children

Part I: Basic Information about Children

Instructions: Please type "√" before the option, or fill in after the colon.

1. Name:
2. Grade:
3. Class:
4. Sex: （1）Male （2）Female
5. Date of birth:
6. Home address:
7. Contact Details for the family:
8. Nationality:（1）Han （2）Hui （3）Dongxiang （4）other
9. Total family size:
10. Number of siblings:（1）0 （2）1 （3）2 （4）3 （5）4 （6）5 or more
11. Father's education level: （1）Elementary school and below （2）Junior high school （3）High school （4）Bachelor degree and above
12. Mother's education level: （1）Elementary school and below （2）Junior high school （3）High school （4）Bachelor degree and above
13. Father's Occupation:
14. Mother's Occupation:
15. Total annual household income（RMB）:（1）Below 10000 （2）10000-19999 （3）

20000-29999　（4）30000-39999　（5）Above 40000

Part II: The Physical Condition of the Children

Instructions: Please type "√" before the option, or fill in the horizontal line.

1. Has the child had symptoms of decreased eating in the last 3 months？

（1）Yes　（2）No

2. Has the child had symptoms of anorexia in the last 3 months？

（1）Yes　（2）No

3. Has the child had symptoms of a partial eclipse in the last three months?

（1）Yes　（2）No

4. Has the child had a fever in the last three months?

（1）Yes　（2）No

5. In the past three months, has the child had any respiratory symptoms such as cough, runny nose, nasal congestion, and sore throat?

（1）Yes　（2）No

6. In the past three months, has the child had any gastrointestinal symptoms such as abdominal pain, constipation, and diarrhea?

（1）Yes　（2）No

7. Has the child had symptoms of nail biting in the last three months?

（1）Yes　（2）No

8. Has the child had symptoms of eating hair in the last three months?

（1）Yes　（2）No

9. Has the child had symptoms of eating wall ash, soil, or gravel in the last three months?

（1）Yes （2）No

10. Has the child had difficulty in concentration in the last three months?

（1）Yes （2）No

11. Has the child had symptoms of memory loss in the last three months?

（1）Yes （2）No

12. Has the child had any other symptoms in the last three months? If yes, please write it down.

（1）Yes,_______________ （2）No

13. Has the child taken any medication in the last three months? If yes, please write it down.

（1）Yes,_______________ （2）No

14. Has the child taken nutritional supplements in the last three months? If yes, please write it down.

（1）Yes,_______________ （2）No

Part III: 24-Hour Dietary Recalls of 3 Consecutive Days

Collect the information on food consumption using the 24-h dietary recall method over three consecutive days (including two weekdays and one weekend day). For children younger than 12 years, the mother or caregiver was asked to report all foods the children consumed. Children over 12 years old recalled food consumption independently.

**Date**	**Time**	**Dining place**	**Food**	**Method of cooking**	**Cooking Materials**	**Material weight (g)**	**Intake (g)**

Date	Time	Dining place	Food	Method of cooking	Cooking Materials	Material weight (g)	Intake (g)

Date	Time	Dining place	Food	Method of cooking	Cooking Materials	Material weight (g)	Intake (g)

Supplementary Table S1. Average intake versus recommended intake for nine food groups.

**Food group**	**Average intake (g)**	**Recommended nutrient intake[1] (g)**
Cereals and potatoes	363.22±8.12	250~400
Vegetables	92.50±5.60	300~500
Fruit	74.35±6.31	200~350
Livestock and poultry meat	29.11±2.24	40~75
Aquatic products	1.36±0.76	40~75
Egg	55.38±2.59	40~50
Milk and milk products	127.55±3.76	300
Soybeans and nuts	3.83±0.18	25~35
Oil	18.11±0.88	25~30

Food intakes are expressed as mean ± SEM. [1]Recommended nutrient intake are adapted from the Dietary Guidelines for Chinese Residents (2016).

Supplementary Table S2. The daily consumption of food groups and nutrients between ZD group and CK group.

	**ZD (n=57)**	**CK (n=120)**	**Total**<br>**（n=177）**	***p***
**Food groups**				
Cereals and potatoes	366.77±13.89	361.54±10.03	363.22±8.12	0.764
Vegetables	94.86±9.91	91.39±6.81	92.50±5.60	0.773
Fruit	81.89±11.08	70.77±7.69	74.35±6.31	0.412
Livestock and poultry meat	27.10±3.95	30.07±2.74	29.11±2.24	0.539
Aquatic products	2.66±2.20	0.76±4.48	1.36±0.76	0.398
Egg	57.55±3.84	54.35±3.37	55.38±2.59	0.566
Milk and milk products	132.94±54.19	125.13±4.37	127.55±3.76	0.333
Soybeans and nuts	4.48±0.43	3.54±0.16	3.83±0.18	0.042*
Oil	16.85±1.16	18.69±1.17	18.11±0.88	0.327
**Nutrients**				
Energy	1316.65±50.72	1322.23±34.50	1320.43±28.45	0.927
Protein	41.23±1.75	40.43±1.14	40.69±0.95	0.699
Fat	45.02±1.82	47.05±1.59	46.40±1.23	0.444
Carbohydrate	186.76±8.44	184.28±5.13	185.08±4.40	0.793
Dietary Fiber	6.33±0.37	5.90±0.18	6.04±0.17	0.310
Vitamin A	265.49±13.50	260.41±9.11	262.05±7.54	0.754

Vitamin D	1.25±0.11	1.22±0.19	1.23±0.13	0.931
Vitamin E	34.01±3.08	35.61±1.98	35.10±1.66	0.655
Vitamin B_1	0.64±0.04	0.62±0.02	0.62±0.02	0.587
Vitamin B_2	0.60±0.03	0.69±0.14	0.66±0.09	0.654
Vitamin C	30.71±3.14	34.51±2.27	33.29±1.84	0.336
Niacin	6.59±0.35	6.54±0.25	6.56±0.20	0.897
Calcium	299.56±10.95	329.68±36.16	319.98±24.75	0.571
Phosphorus	667.09±26.09	651.79±16.39	656.72±13.90	0.609
Potassium	1189.02±57.58	1189.66±48.48	1189.46±37.65	0.994
Sodium	3168.20±225.52	3379.00±158.14	3311.11±129.37	0.448
Magnesium	171.23±8.18	169.71±4.74	170.20±4.14	0.864
Iron	11.94±0.59	12.11±0.39	12.06±0.33	0.796
Zinc	4.87±0.23	4.84±0.14	4.85±0.12	0.924
Selenium	32.24±1.24	31.88±0.87	32.00±0.71	0.814

The intake of food and nutrients are expressed as mean ± SEM. Nutrient units: energy in kcal, protein, fat, carbohydrate, Dietary Fiber and food groups in g, vitamin A an equivalent in µg retinol, selenium and vitamin D in µg, all other nutrients in mg. *p* value was calculated by student's *t* test. $^{*}p < 0.05$.

Supplementary Table S3. Nutrient adequacy ratio of nutrients among participants by zinc statue groups.

NARs	Overall		ZD		CK		*p*
	Mean	SEM	Mean	SEM	Mean	SEM	
Vitamin A	0.523	0.016	0.526	0.026	0.522	0.019	0.888
Vitamin D	0.116	0.007	0.125	0.011	0.111	0.009	0.316
Vitamin E	0.990	0.006	0.994	0.006	0.988	0.008	0.630
Vitamin B_1	0.611	0.016	0.620	0.031	0.607	0.019	0.729
Vitamin B_2	0.574	0.014	0.579	0.025	0.571	0.017	0.803
Vitamin C	0.466	0.022	0.440	0.036	0.478	0.027	0.412
Niacin	0.616	0.017	0.618	0.030	0.616	0.020	0.953
Calcium	0.301	0.008	0.300	0.011	0.301	0.010	0.922
Phosphorus	0.959	0.009	0.964	0.015	0.956	0.012	0.709
Potassium	0.741	0.016	0.754	0.030	0.735	0.019	0.579
Sodium	0.990	0.005	0.995	0.004	0.989	0.008	0.601
Magnesium	0.735	0.016	0.744	0.029	0.731	0.019	0.708
Iron	0.822	0.016	0.816	0.029	0.825	0.019	0.781
Zinc	0.664	0.015	0.675	0.028	0.659	0.018	0.620
Selenium	0.759	0.015	0.778	0.025	0.750	0.019	0.390
MAR	0.658	0.011	0.661	0.019	0.656	0.013	0.801

NAR: Nutrient adequacy ratio. MAR: Mean adequacy ratio. SEM: Standard Error of Mean. *p* value was calculated by student's *t* test.

Supplementary Table S4. Characteristics of zinc deficiency and control children (n = 67).

	**ZD (n=26)**	**CK (n=41)**	***p***
Age, years (mean ± SEM)	8.16 ± 0.19	8.51 ± 0.28	0.312
Sex (females, males)	15females, 11males	25 females, 16 males	0.789
Dietary diversity score	5.77 ± 0.13	5.81 ± 0.09	0.775
Z-score			
Median HAZ score	-0.09	-0.69	0.045*
Median WAZ score	-0.20	-0.53	0.090
Median BMIZ score	-0.23	-0.42	0.077
Blood indices (mean ± SEM)			
Serum zinc (μg/dL)	19.11 ± 3.72	145.7 ± 5.83	<0.001***
IL-6 (pg/mL)	21.62 ± 2.06	17.82 ± 1.83	0.183
TNF-α (pg/mL)	13.80 ± 0.50	7.85 ± 0.85	<0.001***
IL-1β (pg/mL)	24.66 ± 3.63	21.82 ± 2.71	0.526

*p <0.05, ***p <0.001.

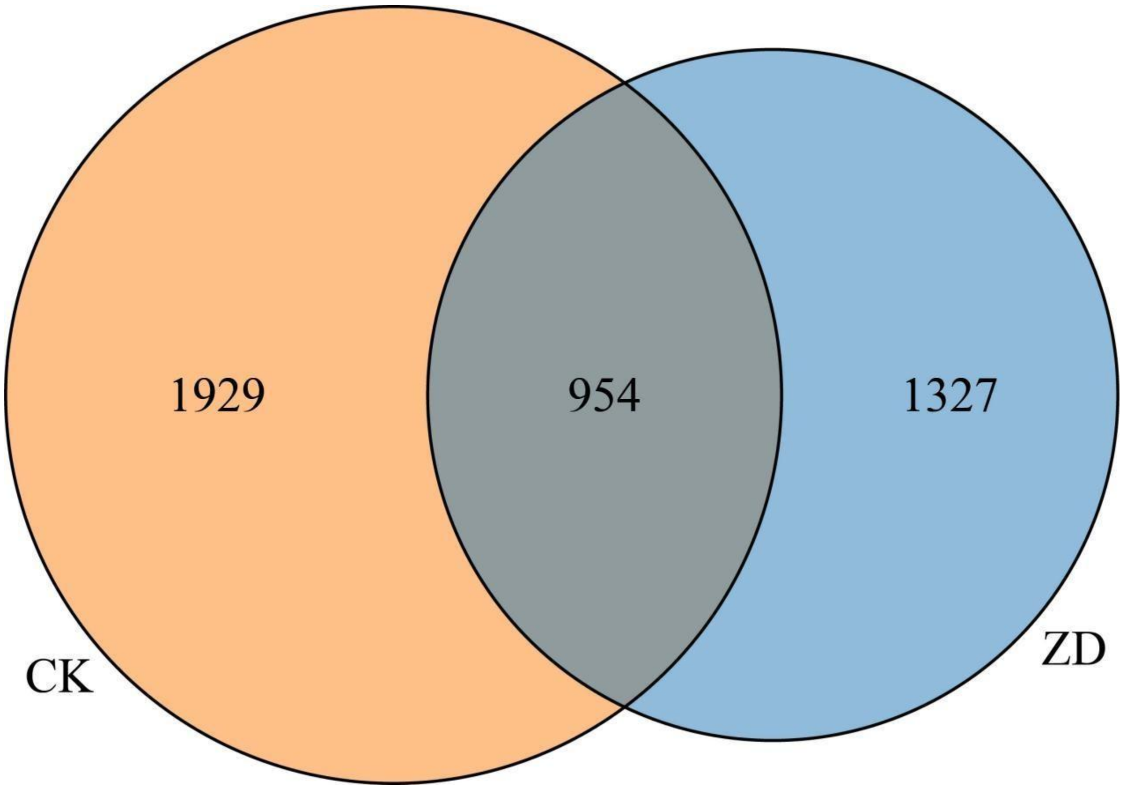

Supplementary Figure S1. Venn diagram of unique and shared ASVs (amplicon sequence variants) in ZD group and CK group.

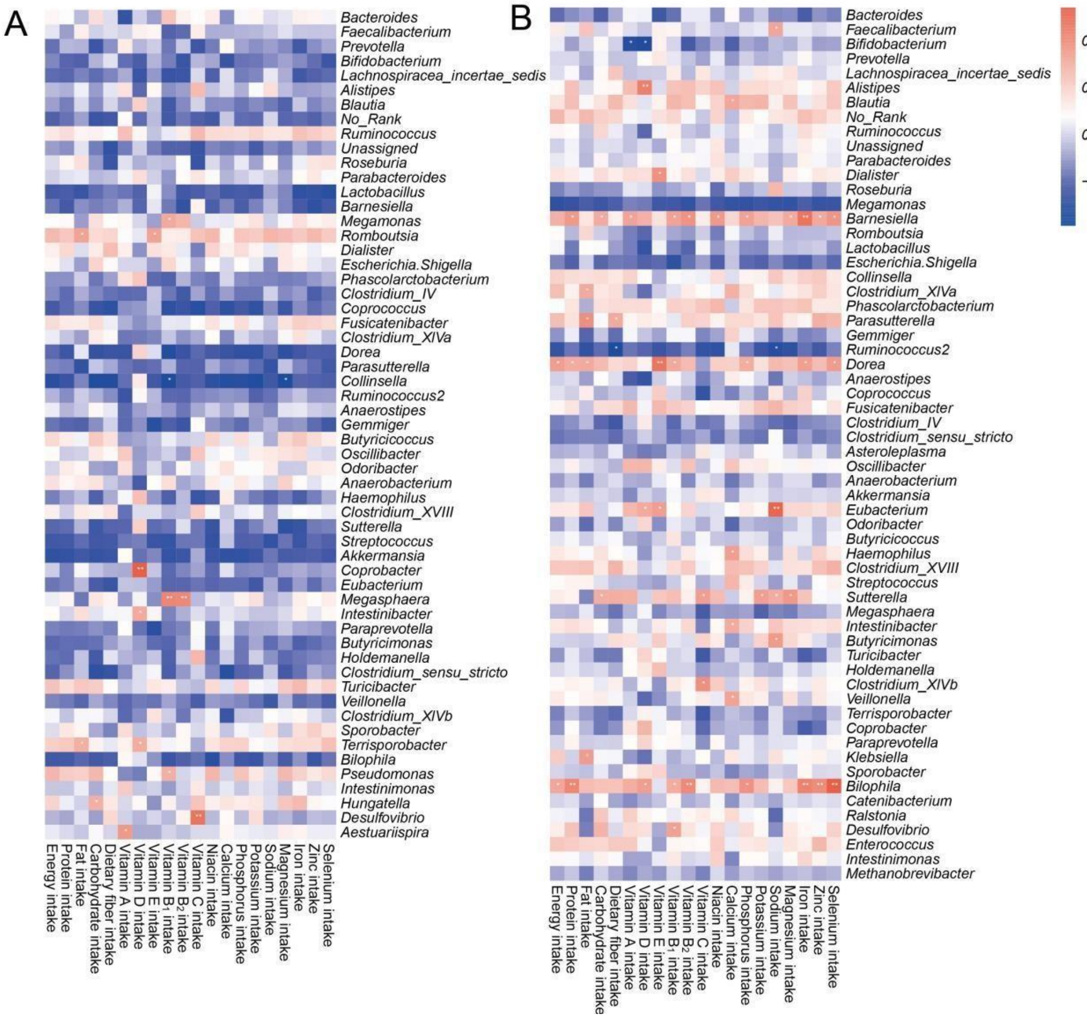

Supplementary Figure S2. The relationship between genus-level bacterial taxa and nutritional profile in **(A)**ZD group and **(B)**CK group. Only those genera with relative abundance >0.05% are shown. Two colors mean positive correlation and negative correlation, respectively.

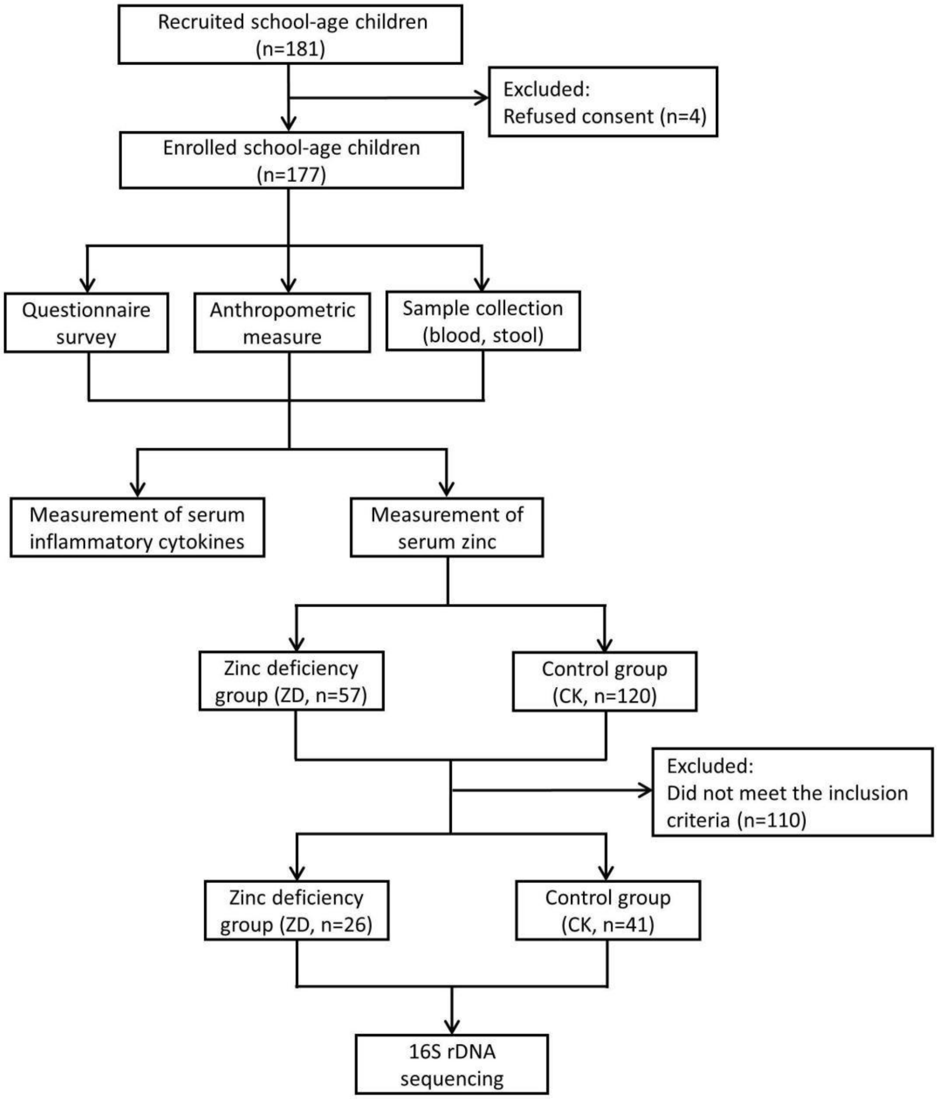

Supplementary Figure S3. Graphical diagram of overall plan in this study.
